# Effects of different customized foot orthoses on isolated subtalar arthrodesis

**DOI:** 10.1186/1757-1146-7-S1-A12

**Published:** 2014-04-08

**Authors:** E Ceccaldi, M Janin

**Affiliations:** 1 Applied Podiatry College, 7 Treguel, 86000 Poitiers, France; 2 Podiatrist, Office, 35 rue Sermonoise, 77380 Combs-la-Ville, France; 3 Podiatrist, PhD, Clinic, 7 Treguel, 86000 Poitiers, France

## Background

Studies describe subtalar and ankle arthrodesis as a factor altering the biomechanics of the foot during walking [1-3]. Furthermore, foot orthoses (FOs) are also recognized for their actions on the dynamics of the foot [4] but not for their actions on an isolated subtalar arthrodesis (ISA). Previous studies have shown that depending on the type of FOs [5] and along the comfort felt by the subject [6], the variations induced by different FOs were significantly different. The aim of this study was to compare the effects of different types of FOs on gait analysis with an ISA. Two subjects with ISA were volunteers for one session of three repeated measures: without FOs (Control), with Classical FOs (FOsC) and with Molded FOs (FOsM). After a clinical examination, these two types of FOs are custom-made including same posting. We compared walking variations through the Latero-Medial Index (LMI) on force platform. Four data’s have been compared: Latero-Medial Force Index (LMFI), Latero-Medial Area Index (LMAI) together with observation of these index at 100ms which correlate with hind foot activity phase during walking [7]. The perception of comfort was evaluated by using previously established footwear comfort measures [6]: 100mm visual analog scale (VAS).

## Results

**Figure 1:**
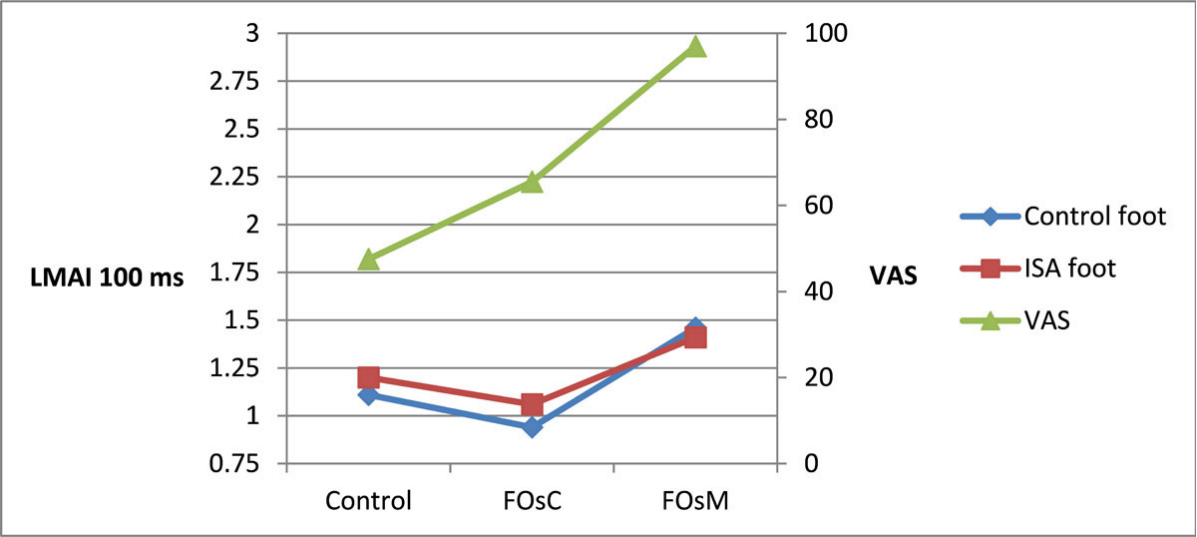
LMAI at 100ms and VAS

**Figure 2:**
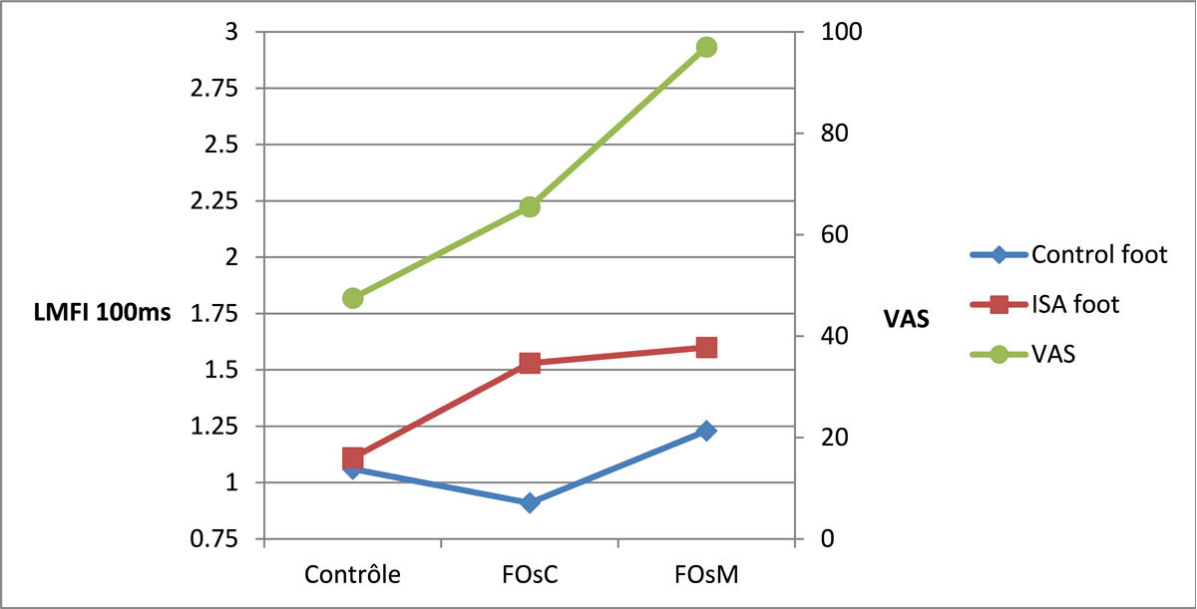
LMFI at 100ms and VAS

Using the VAS, subjects didn’t feel a real comfort in shoes without FOs (VAS=47,5mm). FOs increased VAS (>17,9mm). Thus, FOsM were perceived as significantly more comfortable than FOsC, respectively 97mm and 65,5mm. Dynamics assessment showed the ISA and control foot had same LMI except for the LMAI. LMAI observed at 100ms, FOsC induce pronation on both hind feet while FOsM induce wider lateral contact (Figure 1). LMFI showed FOsC produced an asymmetrical ground reaction force between ISA and control foot. For LMFI at 100ms, the difference increased with FOsC, which induced a higher supination of the ISA and a pronation of the control hind foot. FOsM reduced this difference increasing both supination (Figure 2).

## Conclusions

FOs induced effects on the gait of subjects with ISA depending on orthoses type and parameters observed. The comfort is significantly improved by FOs and much more by FOsM. The data suggests no correlations between linear improvement of VAS and variations of LMI. However, a wider lateral contact and a greater lateral excursion of ground reaction force on the hind foot during walking could induce greater comfort of patients with ISA.
